# World Journal of Emergency Surgery (WJES), World Society of Emergency Surgery (WSES) and the role of emergency surgery in the world

**DOI:** 10.1186/1749-7922-2-3

**Published:** 2007-02-08

**Authors:** Fausto Catena, Ernest E Moore

**Affiliations:** 1 Emergency Surgery and Transplant DPT, St Orsola-Malpighi, University Hospital, Bologna, Italy; 2 Trauma Services, Rocky Mountain Regional Trauma Center, Denver Health Medical Center, Denver, USA; 3 Department of Surgery, Denver Health Medical Center, Denver, USA; 4 Department of Surgery, University of Colorado Health Sciences Center, USA

It has been more than a year since we founded WJES and with great satisfaction we have ascertained how great the interest in this topic is throughout the world in a polyspecialist environment, from ocular surgeons to neurosurgeons to the trauma surgeons who are clearly the protagonists.

As you know we have developed a questionnaire to find out how emergency surgery is organized throughout the world. We have found that there are fundamentally two models, present at the same time and sometime in the same country:

On the one hand there are the emergency surgery departments that burden themselves with almost all of the surgical emergencies that come into the hospital (40% of interviewed surgeons) (excluding hyperspecialist environments) and on the other hand there are hospitals without an emergency surgery department in which surgical emergencies are subdivided among the various general and specialized surgeons.

In some hospitals there are trauma teams (30% of interviewed surgeons) but in others none at all.

Therefore even if worldwide the situation in emergency surgery is so heterogeneous, the work of the various surgeons seems more or less similar regardless of whatever name it goes by, be it emergency surgery, acute care surgery, etc.

80% of interviewed surgeons answer that it would be better to have a more precise understanding of pathologies that they might encounter.

We asked also in our survey a definition of emergency surgery: the answers were based on a time criterion (surgery less than 3–12 hours), on a place criterion (patients that come from emergency department) or on a disease criterion (surgery for trauma).

Obviously it is difficult to define emergency surgery but we think that a possible definition can be: polispecialistic surgery performed for trauma injuries or for non traumatic acute diseases during the same admission in the hospital.

In these last few years we've gone on a world tour to specialist conferences on emergency surgery along with conferences in other specialties, always with an eye toward emergency, in Europe, the USA and the rest of the world.

We also attended world conferences in which we ascertained just how important cultural exchange is on a planetary scale.

The natural consequence of these experiences is to begin considering to found the WSES (World Society of Emergency Surgery).

This doesn't mean yet another scientific society designed to give visibility to certain people or to compete with other societies or associations with an annual Congress.

It is intended to be an instrument.

The objective is to put together all the world experts on emergency surgery every three years in one room for three days (Friday, Saturday, and Sunday). There would be selected lectures and free papers in the same room with a lot of space for discussion.

Also lectures and free papers would be developed as articles for wide publication online at the beginning of the convention on the WJES.

In the end we would put together a video of the conference and make it available on the WJES website.

Every three years the WSES would develop a randomized multicenter study on a theme of general interest as well as guidelines on major themes.

At least this much is clear: we wish to organize the first convention in 2009.

Happy New Year!

